# The impact of nitric oxide on HER family post-translational modification and downstream signaling in cancer

**DOI:** 10.3389/fphys.2024.1358850

**Published:** 2024-03-27

**Authors:** Ciara E. O’Neill, Kai Sun, Sugunapriyadharshini Sundararaman, Jenny C. Chang, Sharon A. Glynn

**Affiliations:** ^1^ Lambe Institute for Translational Research, Discipline of Pathology, School of Medicine, University of Galway, Galway, Ireland; ^2^ Houston Methodist Research Institute, Houston, TX, United States; ^3^ Dr Mary and Ron Neal Cancer Center, Houston Methodist Hospital, Houston, TX, United States

**Keywords:** nitric oxide, cancer, human epidermal growth factor receptor 2, epidermal growth factor receptor, post-translational modification, nitrosation

## Abstract

The human epidermal growth factor receptor (HER) family consists of four members, activated by two families of ligands. They are known for mediating cell–cell interactions in organogenesis, and their deregulation has been associated with various cancers, including breast and esophageal cancers. In particular, aberrant epidermal growth factor receptor (EGFR) and HER2 signaling drive disease progression and result in poorer patient outcomes. Nitric oxide (NO) has been proposed as an alternative activator of the HER family and may play a role in this aberrant activation due to its ability to induce s-nitrosation and phosphorylation of the EGFR. This review discusses the potential impact of NO on HER family activation and downstream signaling, along with its role in the efficacy of therapeutics targeting the family.

## 1 Introduction

Nitric oxide (NO) is a gaseous signaling molecule with a short half-life. In 1987, it was discovered to be the molecule responsible for the ability of endothelium-derived relaxing factor to induce the relaxation of vascular smooth muscle (Ignarro et al., 1987). NO has also been associated with the regulation of other biological systems, including the cardiovascular, nervous, and immune systems (Ignarro, 2000). NO is generated physiologically from oxygen, NADPH, and L-arginine by a family of three nitric oxide synthases: neuronal (nNOS/NOS1), inducible (iNOS/NOS2), and endothelial (eNOS/NOS3). NO synthesis requires the binding of NOS and calmodulin. High levels of intracellular calcium are needed to facilitate the binding of nNOS and eNOS to calmodulin (Nathan and Xie, 1994). This results in the generation of nanomolar levels of NO over a short period of time (seconds/minutes) (Michel and Feron, 1997). However, iNOS has a high affinity for calmodulin, so it does not require high calcium levels, allowing it to produce micromolar NO levels over long periods of time (hours/days). nNOS is predominantly expressed in neurons, where it plays a role in synaptic plasticity and the regulation of blood pressure. The expression of iNOS can be induced in various cell types, following an interaction with stimuli such as lipopolysaccharide or cytokines. The large amounts of NO generated by iNOS have protective effects against pathogens. eNOS is expressed in endothelial cells, where it functions to regulate vasodilation and blood pressure (Förstermann and Sessa, 2012).

NO generated enzymatically by NOS can be rapidly inactivated through conversion to inorganic nitrate (NO_3_) and nitrite (NO_2_^−^). However, NO can also be produced from dietary nitrate and nitrite. Bacteria in the mouth reduce nitrate to nitrite, which travels to the stomach. In the stomach, nitrite undergoes non-enzymatic disproportionation (Lundberg and Weitzberg, 2013). There are various other methods, both enzymatic and non-enzymatic, that convert nitrate to NO, all of which occur at an accelerated rate in acidic and hypoxic conditions, where NOS enzymes may be inactive. NO generated by this pathway is also involved in NO signaling (Lundberg and Weitzberg, 2022).

NO is a key component of redox signaling within biological systems. Its unpaired electron allows it to act as both a reductant and a weak oxidant (Kanner et al., 1991). NO is not stored but simply diffuses to its active site and covalently binds to its targets. These targets include the ions of transition metals such as iron. NO’s interaction with heme facilitates its interactions with hemoglobin and soluble guanylate cyclase (sGC) (Ignarro, 1989). sGC activation by NO mediates NO’s effects on vasodilation and blood pressure and is considered to be classical NO signaling. An alternative signaling mechanism of NO is via s-nitrosation. NO and the related nitrosonium ion (NO^+^) also react with proteins to form S-nitrosothiols (R-SNO), which, as discussed later in this review, allows NO to regulate protein signaling via s-nitrosation.

NO has been found to play a role in various cancers, such as breast, prostate, lung, pancreas, and colon cancers (Fujimoto et al., 1997; Reveneau et al., 1999; Hundley and Rigas, 2006; Stewart et al., 2009; Wang et al., 2016). In cancer, NO can regulate various key components, such as tumor growth, metastasis, and angiogenesis. The effects of NO in cancer are dichotomous and based on the concentration, duration of exposure, NOS isoform, tumor microenvironment, and type of cancer (Burke et al., 2013; Kamm et al., 2019). Low NO concentrations are associated with metastasis and drug resistance, whereas high NO concentrations are linked to increased apoptosis (Vannini et al., 2015).

The human epidermal growth factor receptor (HER) family’s discovery began in the 1960s, when epidermal growth factor (EGF) was first discovered (Cohen, 1965). It was not until the 1980s that the corresponding receptor, epidermal growth factor receptor (EGFR), was successfully cloned and found to be amplified in A431 epidermoid carcinoma cells (Ullrich et al., 1984). It was subsequently discovered that the avian erythroblastosis tumor virus encoded an aberrant variant of EGFR (HER1) (Downward et al., 1984a). This led to the investigation into the oncogenic role of EGFR. Following this, a gene similar to EGFR, which is known as the human epidermal growth factor receptor 2 (HER2) gene, was found to be amplified in a human breast cancer cell line (King et al., 1985). HER2 (human) and *neu* (rodent) are homologs of a growth factor receptor that were discovered independently and found to be oncogenic. Neu was initially found to be homologous to v-erbB (avian erythroblastosis virus), a viral oncogene, and the EGFR (Schechter et al., 1984; 1985). It was later discovered that HER2 also had tyrosine kinase activity (Akiyama et al., 1986). HER2 gene amplification by 2–50 fold was found in ∼30% of breast tumors and was found to be a significant predictor of both overall survival and time to relapse (Slamon et al., 1987). HER3 and HER4 were discovered in the early 1990s (Plowman et al., 1990; 1993). This led to the completion of the HER family as we know it today, consisting of EGFR (HER1, erbB1), HER2 (erbB2, HER2/neu), HER3 (erbB3), and HER4 (erbB4).

HER family members are expressed throughout the body in non-hematopoietic cells. The predominant physiological role of the HER family is in the mediation of cell–cell interactions both during organogenesis and adulthood (Burden and Yarden, 1997). They play a key role in the development of several organ systems, such as the nervous system, heart, skin, lungs, and gastrointestinal tract (Lee et al., 1995; Miettinen et al., 1995). The family has also been shown to play a role in the development of the mammary gland during puberty (Xie et al., 1997; Andrechek et al., 2005). The HER family, particularly EGFR and HER2, has also been implicated in various cancers. Tumors with dysregulated EGFR or HER2 are linked to more aggressive disease and poor clinical outcomes (Slamon et al., 1987; Nicholson et al., 2001).

Classically regulated by ligand binding and dimerization, in this review, we present an alternative form of HER receptor family activation via NO. HER family activation by NO may lead to enhanced tumor HER receptor signaling, with clinical consequences for patient prognosis and therapeutic outcomes. In particular, if NO and HER receptors interact to drive tumor progression, this may represent an opportunity for combination targeting for the treatment of cancer. This review describes the role of the HER receptor family in cancer progression and introduces how these receptors and their downstream signaling pathways are impacted by NO signaling. Understanding the interplay between these diverse processes is key for the future design of dual combinations of HER- and NO-targeting therapeutics.

## 2 HER family receptor structure and activation

HER receptors are type I transmembrane growth factor receptors. They respond to extracellular stimuli by activating intracellular signaling pathways. Structurally, they consist of four domains: an extracellular N-terminal containing two cysteine-rich regions, a transmembrane domain, an intracytoplasmic domain, and a C-terminal tail (Carpenter, 1987).

The HER family is activated by two families of ligands: EGF-related ligands and neuregulins. All of these ligands share an EGF-like domain and three disulfide-bonded loops. The receptor-binding domain tends to be part of a large precursor that undergoes a highly regulated cleavage to release the ligand (Prenzel et al., 1999). Different ligands have differential specificities for each of the family members, as summarized in Table 1. The EGFR is predominantly activated by the EGF, whereas HER3/HER4 is activated by neuregulins. Despite HER2 not having a ligand-binding domain, it is frequently hyperactivated.

**TABLE 1:** HER family ligands.

Ligand	Family	Receptor	Reference
EGF	EGF-like	EGFR	Wada et al. (1990)
Amphiregulin	EGF-like	EGFR	Shoyab et al. (1989)
Transforming growth factor-α	EGF-like	EGFR	Marquardt et al. (1984)
Epigen	EGF-like	EGFR	Strachan et al. (2001)
Betacellulin	EGF-like	EGFR	Shing et al. (1993)
Connective tissue growth factor	EGF-like	EGFR	Rayego-Mateos et al. (2013)
Heparin-binding EGF	EGF-like	EGFR and HER4	Higashiyama et al. (1991)
Epiregulin	EGF-like	EGFR and HER4	Toyoda et al. (1995)
No known direct ligand	N/A	HER2	Slamon et al. (1989)
NRG-1^a^	Neuregulins	HER3 and HER4	Riese et al. (1995)
NRG-2	Neuregulins	HER3 and HER4	Riese et al. (1995), Carraway et al. (1997)
NRG-3	Neuregulins	HER4	Riese et al. (1995), Zhang et al. (1997)
NRG-4	Neuregulins	HER4	Riese et al. (1995), Harari et al. (1999)

^a^ Neuregulin-1 (NRG-1) is also known as the neu differentiation factor, heregulin, acetylcholine receptor-inducing activity, and glial growth factor.

**FIGURE 1:**
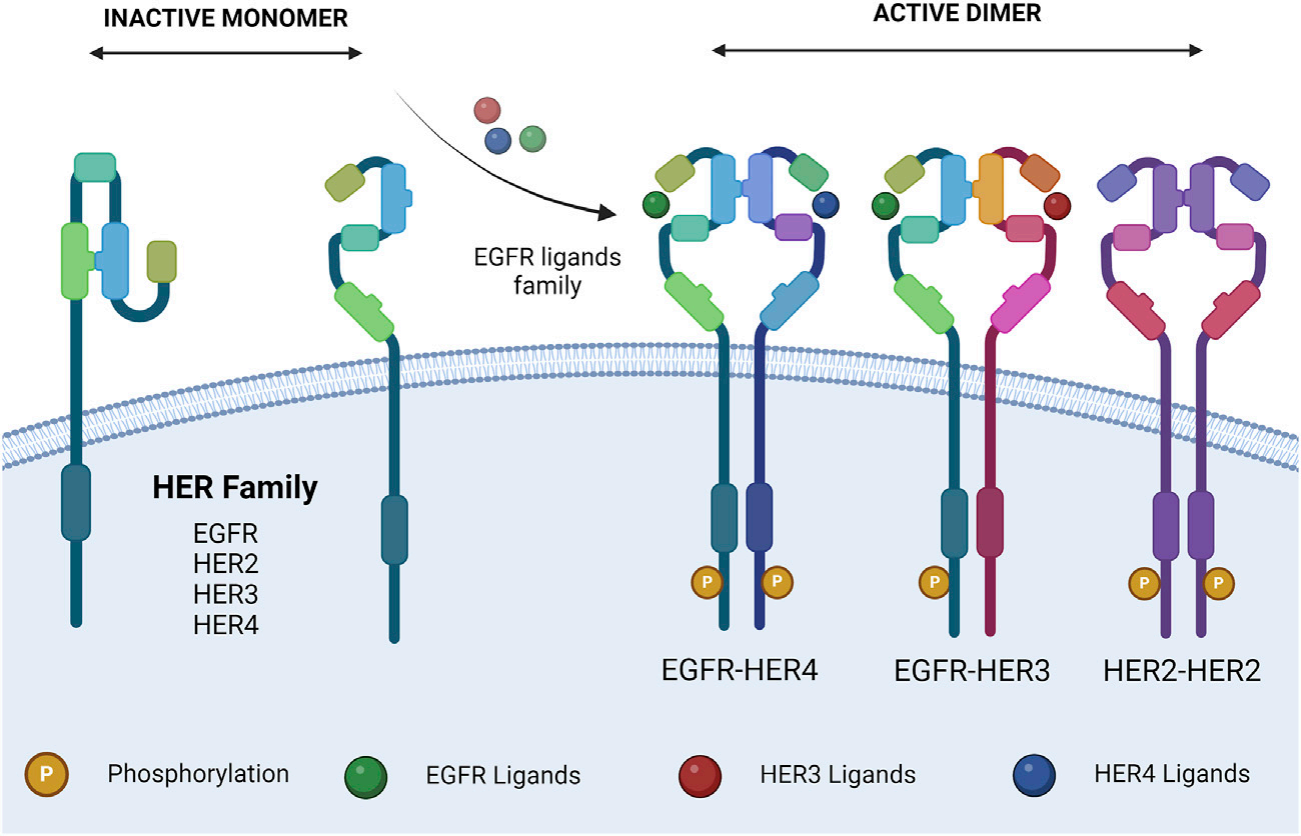
HER family dimerization. HER family ligand interactions, and subsequent dimerization. Ligands are described in Table 1. The dimers shown are representative. Created with BioRender.com.

Upon extracellular binding with their given ligand, the extracellular domain undergoes a conformational change from a closed inhibited state to an open active state that promotes dimerization (Figure 1) (Lemmon et al., 1997). Dimer partner selection is an important factor in determining the downstream signaling pathways activated by receptor activation. There is a hierarchy of interactions between the transmembrane domains of the four receptors, ranging from non-interactive pairs to strong dimerization. The preferred dimer partner was found to be HER2, with HER3 ranking next ahead of EGFR (Duneau et al., 2007). HER2 has the strongest kinase activity; therefore, dimers containing HER2 have stronger downstream signaling (Graus-Porta et al., 1997). The HER3–HER4 heterodimer is the least favored thermodynamically and could not be induced by ligand binding (Tzahar et al., 1996). Despite this, HER4 signaling has been documented in breast and nervous system development (Tidcombe et al., 2003).

HER2 exists in a constitutively active state (Garrett et al., 2003) and lacks the ability to bind a ligand; therefore, the ability of ligand binding to induce HER2 signaling is dependent on a heterodimeric partnership (Sliwkowski, 2003). On the other hand, HER3 is missing an ATP-binding site within its catalytic domain, preventing its kinase activity (Sierke et al., 1997), meaning that HER3’s downstream signaling is also dependent on a heterodimeric partnership (Kim et al., 1998). Despite being incomplete individually, the HER2–HER3 heterodimer is the most active of the family and is essential for various processes.

The ligand(s) involved in receptor activation can also play a role in dimer selection. In dimers where two ligands can be present (e.g., EGFR–HER3), they are more stabilized by neuregulin than the EGF (Tzahar et al., 1996). Dimer formation allows for the activation of the kinase domain and trans-auto-phosphorylation of the intracellular domain. Each member of the HER family has different C-terminal sites, which becomes trans-auto-phosphorylated upon dimer formation. This allows for the subsequent docking of signaling molecules, triggering downstream signaling and the various biological effects associated with HER family signaling (Olayioye et al., 2000).

There are two pathways involved in the post-activation processing of the HER family. The EGFR undergoes endocytic degradation, whereas HER2, HER3, and HER4 undergo endocytic recycling (Baulida et al., 1996). When the EGFR dimerizes with HER2, such as in HER2 overexpression, it is redirected down the endocytic recycling pathway. This results in increased levels of EGFR on the cell membrane, along with longer and more potent signaling activity (Lenferink et al., 1998; Waterman et al., 1998; Hendriks et al., 2003). Overall, HER2 is the least frequently inactivated member of the HER family. HER2-containing dimers can trigger downstream signaling for prolonged periods by evading signal attenuation. This leads to increased MAPK and c-Jun activation in HER2-overexpressing cells, following treatment with EGFR or HER3 ligands (Karunagaran et al., 1996).

## 3 Interaction of NO with HER family signaling

HER family signaling varies in complexity from organism to organism. In *C. elegans*, signaling is driven by a single ligand and receptor; in *Drosophila*, this increases to four ligands and one receptor (Lacenere and Sternberg, 2000), whereas in mammals, there are at least 12 ligands and 4 receptors. The extent of the HER family in mammals compared to other animals is believed to result from functional differentiation, requiring all members of the family to interact in their various heterodimers to carry out different functions downstream. The fact that HER2 and HER3 are functionally incomplete facilitates this concept.

**FIGURE 2:**
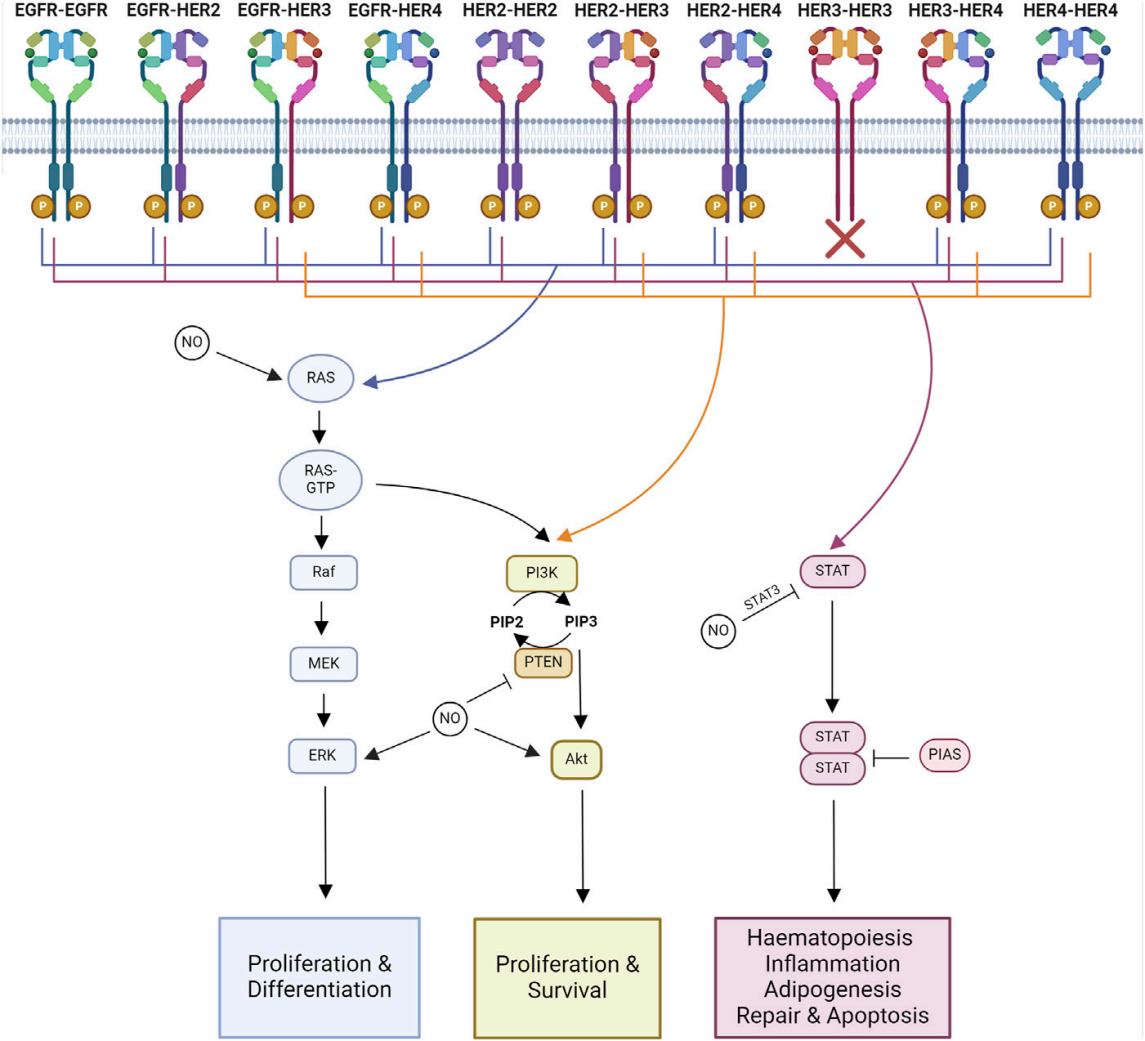
Major signaling pathways impacted by NO-HER family interactions. Major downstream signaling pathways activated by each HER family dimer pair and known interactions of NO. Ras-MAPK signaling regulates proliferation and differentiation. PI3K-Akt signaling regulates proliferation and survival. STAT signaling regulates hematopoiesis, inflammation, adipogenesis, repair, and apoptosis. Created with BioRender.com.

The specific tyrosine phosphorylation residues of each member of the HER family control the binding ability of downstream signaling molecules. Some molecules are common among multiple members of the family, such as Grb2 and Shc, whereas others are more specific, like Cbl, which only binds to the EGFR (Levkowitz et al., 1996). Therefore, each of the 10 HER family dimers exhibits differences in the downstream signaling pathways they activate (Figure 2).

Homodimers tend to be less able to stimulate proliferation compared to heterodimers. Within the heterodimers, those containing HER2 are the most active, while those containing HER3–HER4 are the least active, linking into the hierarchal process of dimer formation. HER2’s highly active signaling is due to its ability to decrease the rate of NRG and EGF dissociation from their receptors alongside HER2’s slow endocytosis rate, leading to the amplification and increased duration of signaling (Graus-Porta et al., 1995; Karunagaran et al., 1996). HER2 overexpression alters the profile of HER family dimers by increasing the formation of HER2–EGFR and HER2–HER3 heterodimers (Karunagaran et al., 1996; Hendriks et al., 2003).

The functional results of HER family signaling are varied and include migration, mitosis, adhesion, differentiation, and apoptosis. The end result is dependent on the cell type and the ligand and receptor combinations (Yarden and Sliwkowski, 2001). The core pathways downstream of HER family activation are Ras-MAPK, PI3K-Akt, and JAK-STAT signaling.

### 3.1 Ras-MAPK

The Ras-MAPK pathway controls proliferation and differentiation (Iwakura and Nawa, 2013). MAPK signaling activated through Ras and Shc is a downstream target of all HER family dimers (Yarden and Sliwkowski, 2001).

Activated HER family dimers, once phosphorylated at an appropriate residue, become associated with Shc and Grb2. Grb2 and Shc both have phospho-tyrosine-binding sites in all members of the HER family, as outlined above. Grb2 or Shc binding then recruits SOS, a Ras-guanine nucleotide exchange factor, leading to the activation of Ras and setting off the kinase cascade that activates Raf, MEK, and ERK (Iwakura and Nawa, 2013). ERK1/2 further interacts with various molecules that promote cell division (Yamamoto et al., 2006).

In addition to direct NO action on HER receptors, Ras s-nitrosation modulates the effects of NO on the Raf/MEK/ERK and PI3K/Akt pathways (Pervin et al., 2007). Ras is aberrantly activated in breast cancers overexpressing EGFR or HER2 (von Lintig et al., 2000). S-nitrosation of Ras has been linked to metastasis through MAPK-dependent Ets-1 activation (Marshall and Foster, 2012). NO also phosphorylates ERK1/2 and increases cell migration in an EGFR-ERK1/2-dependent manner in triple-negative breast cancer (Garrido et al., 2017). NO induces tumor growth through MEK1/2 and ERK1/2 phosphorylation, potentially due to a combination of HER receptor and Ras modifications (Rice et al., 2010; Sen et al., 2013; Chen et al., 2018). In colon cancer, NO’s phosphorylation of ERK1/2 also leads to the upregulation of MMP-2 and MMP-9 expression (Babykutty et al., 2012).

### 3.2 PI3K-Akt

The PI3K-Akt signaling pathway regulates cell growth and anti-apoptotic signaling (Iwakura and Nawa, 2013). The PI3K pathway is activated downstream of most HER dimer pairs. However, the extent and kinetics of the activation are varied. This is due to the ability of PI3K to directly interact with HER3 and HER4, whereas it can only interact indirectly with EGFR and HER2 (Soltoff and Cantley, 1996).

HER3 plays a major role in the activation of pro-survival signaling through PI3K due to its six binding sites for p85, PI3K’s regulatory subunit, whereas HER4 only has one p85-binding site.

Aberrant NOS expression is linked to the ability of oncogenic PI3K/Akt signaling to induce inflammation and immunosuppression (Villegas et al., 2018). In breast cancer, Akt phosphorylation and iNOS expression are strongly correlated (Smeda et al., 2018). NO is capable of inducing Akt phosphorylation and tumor growth (Ridnour et al., 2012; Sen et al., 2013). The threshold for Akt phosphorylation is 100 nM of NO (Thomas et al., 2008). NO also protects against H_2_O_2_-induced cell death neuroblastoma through Akt phosphorylation (Yoo et al., 2018). In ovarian cancer, GSNO was found to induce the s-nitrosation of EGFR and Akt but decrease Akt phosphorylation (Giri et al., 2014). In gastric cancer, NO has an anti-proliferative effect and also inhibits Akt phosphorylation (Sang et al., 2011). In ER-negative breast cancer with high levels of iNOS, Akt phosphorylation at S^473^ is present in 87%–89% of tumors, whereas abundant phosphorylation of Akt at T^308^ is linked with co-expression of iNOS and COX2. Downstream of Akt, phosphorylated forms of BAD and caspase 9 were also more abundant in tumors co-expressing iNOS and COX2, showing an association with activation of the Akt signaling pathway. Additionally, Ras-MAPK and/or PI3K-Akt signaling are required for NO to induce COX2 expression (Basudhar et al., 2017).

PTEN, the negative regulator of PI3K signaling, is also highly regulated by NO. S-nitrosation of PTEN at C^83^ results in the inhibition of its enzymatic activity and the induction of its degradation via NEDD4-1-mediated ubiquitination (Kwak et al., 2010; Numajiri et al., 2011; Ohno et al., 2015). S-nitrosation of PTEN has been shown to occur via trans-nitrosation in the brain, with s-nitrosated DJ-1 donating its NO group to PTEN (Choi et al., 2014). nNOS activity has been shown to induce s-nitrosation of PTEN, resulting in the activation of Akt/mTOR signaling and inhibition of autophagy in nasopharyngeal carcinoma (Zhu et al., 2019). S-sulfhydration of PTEN at C^71^ and C^124^ occurs endogenously and prevents s-nitrosation of PTEN (Ohno et al., 2015). Therefore, s-sulfhydration of PTEN allows it to inhibit PI3K signaling, whereas s-nitrosation of PTEN allows for increased PI3K signaling. iNOS expression and the resulting increase in PI3K signaling have been associated with poor clinical outcomes in a subset of melanoma patients expressing PTEN (Ding et al., 2021).

### 3.3 JAK-STAT

JAK/STAT signaling is involved in hematopoiesis, tissue repair, inflammation, apoptosis, and adipogenesis (Bach et al., 1996; Stephens et al., 1996; Fulda and Debatin, 2002; Owen et al., 2019; Jaiswal et al., 2023). JAKs are known to be recruited to activated EGFR, where they become transphosphorylated (Iwakura and Nawa, 2013). Phosphorylated JAK is active and further phosphorylates the receptor, forming a STAT docking site. When STAT binds, it is phosphorylated by JAK, triggering the disassociation of active STAT. Active STAT then forms dimers capable of translocation to the nucleus, where they regulate gene transcription (Bharadwaj et al., 2020).

EGFR activation leads to STAT1 activation and the formation of STAT1 and STAT3 complexes with JAK1 and JAK2 (Andl et al., 2004). JAK2’s phosphorylation of the EGFR has been linked to the activation of Ras-MAPK signaling (Wong et al., 1992; Yamauchi et al., 1997). The EGFR can interact with all STATs, with the exception of STAT6 (Erdogan et al., 2022). In breast cancer, HER2 overexpression has been linked to STAT3 expression and a HER2-STAT3 signaling network (Diaz et al., 2006; Duru et al., 2012). NRG-1, the ligand for HER3 and HER4, can activate JAK/STAT signaling through JAK3, STAT3, and STAT5 in an HER2–HER3-dependent manner. This has been linked to an induction of proliferation (Liu and Kern, 2002). HER4 becomes truncated by γ-secretase, forming a soluble intracellular domain with signaling activity (Ni et al., 2001). This truncated HER4 acts as a chaperone for the translocation of STAT5A to the nucleus (Long et al., 2003).

Both STAT3 and its repressor, Pias3, have been reported to undergo s-nitrosation. S-nitrosation of STAT3 results in a decrease in its phosphorylation at Y^705^ (Giri et al., 2014). S-nitrosation of Pias3 promoted its degradation (Qu et al., 2007). High concentrations of NO have been reported to induce apoptosis in ovarian cancer. This has been linked to a decrease in STAT3 and Akt phosphorylation (Kielbik et al., 2013). The reduction in phosphorylation may be due to the reported inverse relationship between s-nitrosation and phosphorylation in STAT3 and Akt (Giri et al., 2014).

## 4 HER family post-translational modifications

**FIGURE 3:**
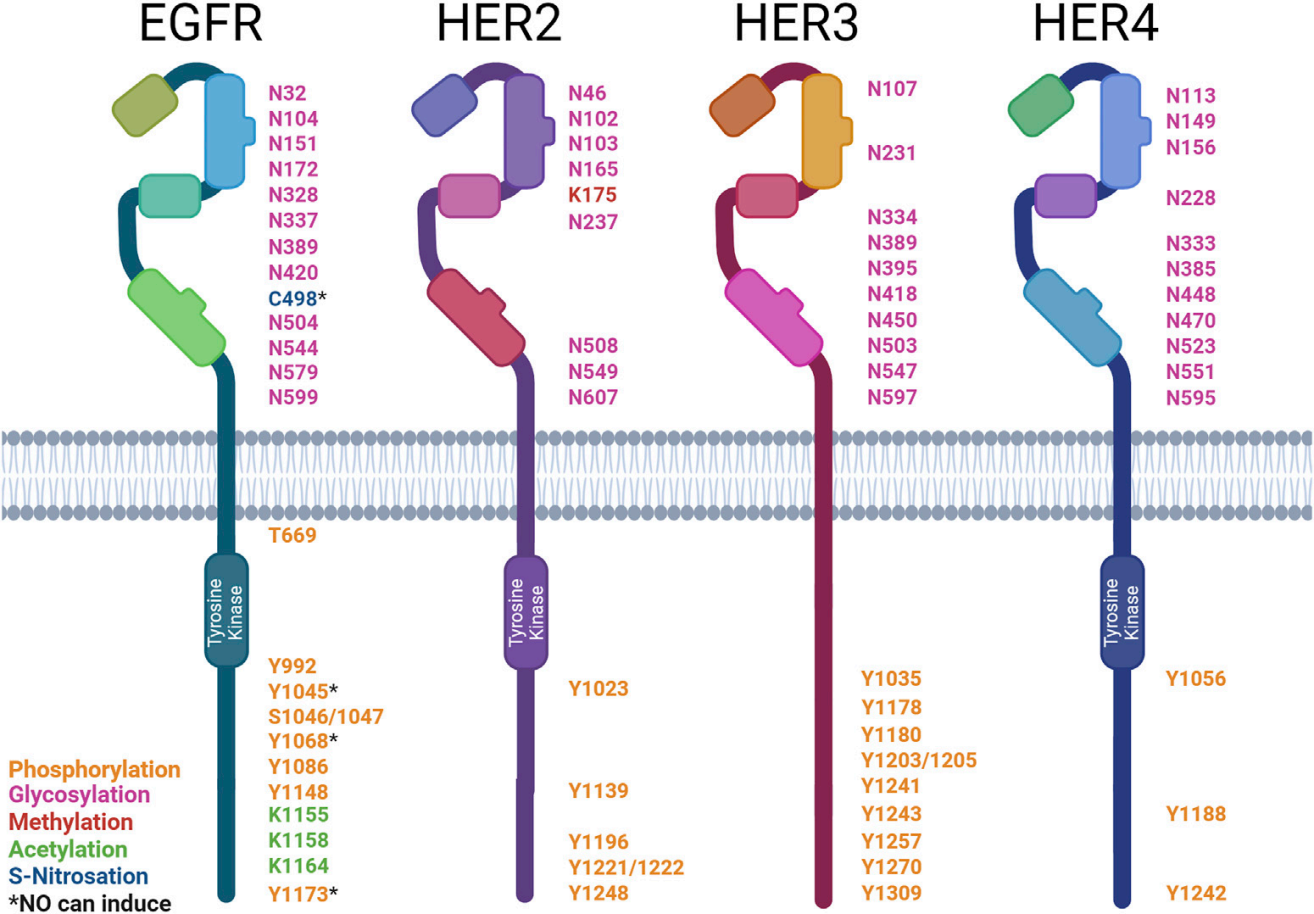
Post-translational modification sites of HER family receptors. HER family receptor structures indicating the sites of post-translational modification (PTM): asparagine (N), cysteine (C), lysine (K), serine (S), tyrosine (Y), and threonine (T). The types of modification are color-coded: phosphorylation (orange), glycosylation (pink), methylation (red), acetylation (green), and S-nitrosation (blue). * denotes PTMs that can be induced by NO. Created with BioRender.com.

The HER family undergoes extensive post-translational modifications (PTMs) (Figure 3). The PTM of proteins involves the addition of functional groups, cleavage, or degradation of translated proteins by various enzymes. This review will focus on the addition of functional groups. S-nitrosation is a PTM mediated by NO. Other common PTMs include phosphorylation, glycosylation, acetylation, methylation, and ubiquitination, all of which can be regulated by NO signaling. PTMs can be classified by the amino acids they modify, the type of enzyme, and the reversibility of the modification (Walsh et al., 2005). The role of PTMs is to regulate protein function. This can be done allosterically or through the creation of binding sites to facilitate protein–protein interactions (Seet et al., 2006).

### 4.1 Phosphorylation

The phosphorylation of proteins is the most common PTM and plays a role in the regulation of almost all cellular processes. Kinases are the enzymes that mediate the addition of a phosphate group to a protein; they catalyze the transfer of γ-phosphate from ATP to an amino acid. The most commonly phosphorylated amino acids are serine, threonine, and tyrosine. On the other hand, phosphatases remove phosphate groups from proteins, further allowing for the regulation of cellular functions (Ubersax and Ferrell Jr, 2007).

The HER family functions as kinases and undergoes trans-phosphorylation in dimer pairs, as previously discussed. Each member of the HER family has different C-terminal tyrosine sites that become trans-auto-phosphorylated. These sites act as docking sites for SH2 and PTB domains on various enzymes and adapter proteins involved in downstream signaling. Protein microarrays have been used to screen all SH2 and PTB domains within the human genome for interactions with the HER family (Jones et al., 2006).

EGFR’s activation leads to the autophosphorylation of six tyrosine residues (Downward et al., 1984b; Hsuan et al., 1989; Margolis et al., 1989; Walton et al., 1990). EGFR’s phospho-tyrosines then interact with various downstream signaling molecules (∼7.2 proteins per site) (Jones et al., 2006). Some of these downstream signaling molecules are Shc (Y^1173^ and Y^1148^), PLCγ (Y^1173^ and Y^992^), SHP1 (Y^1173^), Grb2 (Y^1068^ and Y^1086^), and Cbl (Y^1045^) (Rotin et al., 1992; Batzer et al., 1994; Okabayashi et al., 1994; Okutani et al., 1994; Keilhack et al., 1998; Sakaguchi et al., 1998; Chattopadhyay et al., 1999; Levkowitz et al., 1999). The EGFR phosphorylates PLCγ at Y^771^ and Y^1254^ (Wahl et al., 1990). This increases the phospholipase activity required for EGF-associated cell motility (Xie et al., 1998). Cbl docking facilitates the ubiquitination and subsequent degradation of the EGFR (Waterman et al., 2002). Shc and Grb2 interactions allow for the activation of MAPK/ERK signaling (Rojas et al., 1996; Sakaguchi et al., 1998). NO signaling via the EGFR has been well-documented in breast cancer, with high levels of iNOS expression correlated with EGFR Y^1173^ phosphorylation in ER-negative patient samples. NO has also been shown to rapidly induce EGFR Y^1045^, Y^1068^, and Y^1173^ phosphorylation in triple-negative breast cancer cells (Glynn et al., 2010; Garrido et al., 2017). EGFR phosphorylation at Y^1173^ by NO has also been documented in lung cancer (Lee et al., 2008). In contrast, NO has also been shown to inhibit the phosphorylation of the EGFR by the EGF, causing a downstream reduction in ERK phosphorylation in gastric cancer cells through the activation of type II cGMP-dependent protein kinase (PKG II), demonstrating both pro- and anti-stimulatory effects on the EGFR (YAO et al., 2015).

HER2 activation leads to the autophosphorylation of five tyrosine residues (Hazan et al., 1990; Segatto et al., 1990). These phospho-tyrosines then interact with various downstream signaling molecules (∼17 proteins per site) (Jones et al., 2006). Similarly to the EGFR, HER2’s phospho-tyrosines interact with Shc’s PTB domain (Y^1196^ and Y^1248^), Shc’s SH2 domain (Y^1248^ and Y^1221/2^), and Grb2 (Y^1139^) (Ricci et al., 1995). HER2 also interacts with Chk (Y^1248^) (Zrihan-Licht et al., 1998). The HER2-pY^1112^ site was found to specifically regulate HER2 ubiquitination, the HER2-pY^1196^ site is involved in the regulation of cell motility, and the HER2 pY^1248^ site regulates both migration and proliferation (Li et al., 2016).

HER3 activation leads to the autophosphorylation of nine tyrosine residues These phospho-tyrosines then interact with various downstream signaling molecules (∼8.8 proteins per site) (Jones et al., 2006). These phospho-tyrosines then interact with various downstream signaling molecules, such as Shc (Y^1309^), PI3K’s p85 subunit (Y^1035^, Y^1178^, Y^1203^, Y^1241^, Y^1257^, and Y^1270^), and Grb7 (Y^1180^) (Prigent and Gullick, 1994; Fiddes et al., 1998). HER3 differs from EGFR and HER2 in the absence of a Grb2- and PLCγ-binding site and the presence of PI3K-binding sites (Prigent and Gullick, 1994; Songyang et al., 1995).

HER4 activation leads to autophosphorylation of three tyrosine residues. These phospho-tyrosines then interact with various downstream signaling molecules (∼2.3 proteins per site) (Jones et al., 2006). These phospho-tyrosines then interact with various downstream signaling molecules, such as Shc (Y^1242^ and Y^1188^) and PI3K’s p85 subunit (Y^1056^) (Cohen et al., 1996; Elenius et al., 1999).

Little is known about the impact of NO on the activation status of HER2, HER3, or HER4. Given NO’s ability to regulate the EGFR and other tyrosine kinase inhibitors, there is potential for NO to also regulate the other members of this receptor family in a similar fashion. Indeed, disruption in the equilibrium of tyrosine phosphorylation has been linked to many disease states, including cancer (Hunter, 2009). Aberrant phosphorylation of the HER family occurs in various cancers, including breast, lung, and brain cancers (Slamon et al., 1987; Moscatello et al., 1995).

Phosphatases form the other piece of the puzzle that regulates the equilibrium of phosphorylation. They act by removing the phosphate groups added by kinases. Therefore, they act as antagonists to kinase receptor signaling and play a tumor-suppressive role (Li et al., 1997; Li and Sun, 1997). Two PEST-containing phosphatases, PTPN12 and BDP1 (PTPN18), have been identified as potent negative regulators of HER2 signaling. BDP1 dephosphorylates HER2 at pY^1112^, pY^1196^, and pY^1248^. On the contrary, PTPN12 acts on EGFR pY^1148^ and HER2 pY^1112^, pY^1196^, pY^1221/1222^, and pY^1248^ (Li et al., 2016). PTPN12 acts as a potent suppressor of proliferation, transformation, and metastasis through the inhibition of EGFR/HER2 signaling in mammary epithelial cells (Sun et al., 2011). NO has been shown to increase PTPN12 activity via cGMP signaling; therefore, this is a possible mechanism for NO to downregulate EGFR/HER2 signaling. This also demonstrates a precedent for NO to regulate phosphatases (Lin et al., 2003).

The HER family is also phosphorylated at serine and threonine residues. TNFα has been shown to induce the phosphorylation of EGFR S^1046/7^ and T^669^ through the activation of the ERK and p38 MAPK pathways (Noguchi et al., 2013). The phosphorylation of S^1046/7^ is linked to the internalization of the EGFR, whereas phosphorylation at T^669^ has been shown to suppress the constitutive tyrosine phosphorylation in EGFR homo- and heterodimers (Sato et al., 2013).

### 4.2 S-nitrosation

S-nitrosation is the reversible addition of NO to cysteine via sulfur, forming an S-NO bond known as S-nitrosothiol (Gaston et al., 2003). S-nitrosation occurs spontaneously upon the generation of NO by the NO synthase enzymes (NOS). Once a protein becomes s-nitrosated, it can trans-nitrosate other proteins it interacts with, thus amplifying the signal (Mitchell and Marletta, 2005; Kornberg et al., 2010; Nakamura and Lipton, 2013). S-nitrosation can also be regulated by nitrosylase and denitrosylase enzymes (Kornberg et al., 2010; Anand et al., 2014). NO is known to interact with over 3,000 proteins, predominantly via s-nitrosation (Mnatsakanyan et al., 2019). S-nitrosation of cysteines can alter protein activity and, therefore, cellular signaling (Park, 1988). Alterations in protein s-nitrosation have been associated with various disease states, including cancer (Foster et al., 2009).

S-nitrosation of the EGFR at C^498^ has been shown to activate the receptor. Downstream of EGFR s-nitrosation, oncogenic signaling pathways, including c-Myc, Akt, STAT3, and β-catenin, are activated in breast cancer. The NO concentration threshold for EGFR activation is between 200 and 300 nM; therefore, an autoxidation product of NO such as N_2_O_3_ is considered to be responsible (Switzer et al., 2012). Given that NO can s-nitrosate the EGFR and impact its signaling, it is important that future studies also examine whether NO plays a similar role on the other HER family members.

### 4.3 Other PTMs

The HER family undergoes various other post-translational modifications, such as glycosylation, acetylation, methylation, and ubiquitination. However, any role of NO in the formation of these modifications has yet to be determined.

The extracellular domain of the HER family receptors undergoes extensive post-translational glycosylation, which regulates their ability to bind ligands, form dimers, and activate downstream signaling. Glycosylation has also been shown to modulate the response to anti-HER2 therapeutics. α2,6-Sialylation of HER has been associated with increased resistance to trastuzumab and increased Akt and ERK phosphorylation despite reduced HER2 phosphorylation (Liu et al., 2018). However, no direct link between NO and HER family glycosylation has been reported. NO has been found to increase N-glycan, α2,6-sialylation, and O-GlcNAcylation levels in neuroblastoma (Van de Wouwer et al., 2011). In plants, a link was also found between s-nitrosation and N-glycosylation through the co-substrate thioglucoside glucohydrolase-2 (Du et al., 2019).

Acetylation of the EGFR has been shown to affect tyrosine phosphorylation. K-deacetylase inhibition induces EGFR phosphorylation (Zhou et al., 2006; Song et al., 2011). Receptor turnover and endocytosis are also regulated (Gao et al., 2010; Goh et al., 2010). Although no acetylation sites have been reported on HER2, HER3, or HER4, their structural similarity to the EGFR makes acetylation likely. Using MusiteDeep, a deep learning framework (Wang et al., 2020), we predicted the following acetylation sites: EGFR—K^229^, K^238^, K^262^, K^438^, K^807^, K^928^, K^969^, K^981^, K^984^, K^997^, and K^1283^; HER2—K^753^ and K^854^; HER3—K^177^, K^383^, K^602^, K^705^, and K^926^; HER4—K^751^, K^852^, K^1223^, and K^1269^. NO has not yet been shown to induce EGFR acetylation. However, NO has been associated with the modulation of histone acetylation by s-nitrosation of histone deacetylases (Colussi et al., 2008).

The methylation of HER2 at K^175^ by SMYD3 results in increased receptor phosphorylation and formation of HER2–HER2 homodimers (Yoshioka et al., 2017). There is neither any literature report on the direct methylation of EGFR, HER3, or HER4, nor on the ability of NO to induce acetylation of the HER family. However, NO plays a role in DNA and histone methylation (Socco et al., 2017). Although no methylation sites have been reported on EGFR, HER3, or HER4, their structural similarity to HER2 makes acetylation likely. We predicted the following methylation sites: EGFR—K^1047^; HER3—R^525^, K^959^, R^1042^, and R^1089^; and HER4—K^2^ and K^935^ (Wang et al., 2020).

All HER family receptors undergo ubiquitination, a regulator of their degradation. Ubiquitin-specific protease 2a (USP2a) inhibits EGFR’s endocytosis and subsequent degradation, therefore increasing its stability (Liu et al., 2013). PTPN18 ubiquitinates HER2 at K^48^, triggering rapid proteasomal degradation. Conversely, PTPN’s de-phosphorylase activity at Y^1112^ inhibits the trafficking of HER2 to the lysosome, preventing degradation (Wang et al., 2014). HER3’s ubiquitination is mediated by Nrdp1, an E3 enzyme. This regulates receptor expression levels. In breast cancer, Nrdp1 expression is lost, facilitating increased HER3 expression and downstream signaling (Printsev et al., 2014). HER4 is polyubiquitinated through an interaction with the WW domains of the E3 enzyme, AIP4/Itch (Omerovic et al., 2007). NO has not been reported to affect the ubiquitination of the HER family but has been found to interact with ubiquitination machinery within the cell. NO alters ubiquitination through the inhibition of ubiquitin E1 (Kitagaki et al., 2009) and s-nitrosation of E2 and E3 enzymes (Qu et al., 2007; Bailey et al., 2018).

## 5 Targeting the NO-HER family axis in tumors

As all HER family dimers activate the pro-proliferative Ras-MAPK pathway, the family is frequently dysregulated in cancer. Aberrant phosphorylation of the family has been documented in various cancers, including breast, lung, and brain cancers (Slamon et al., 1987; Moscatello et al., 1995), making the family a well-exploited drug target in cancer (Table 2). The use of therapeutics targeting NO and HER family activity is discussed below, along with interactions between NO signaling and responses to HER family-targeted therapeutics.

**TABLE 2:** EMA-approved HER family-targeted therapeutics.

Drug	Class	Target	Indications	Reference
Gefitinib	TKI	EGFR	EMA-approved—NSCLC (advanced/metastatic, EGFR-activating mutations)	EMA (2023d)
Erlotinib	TKI	EGFR	EMA-approved—NSCLC (advanced/metastatic, EGFR-activating mutations or prior failed chemotherapy) and pancreatic cancer (metastatic, in combination with gemcitabine)	EMA (2023h)
Afatinib	TKI	EGFR	EMA-approved—NSCLC (advanced/metastatic, EGFR-activating mutations or prior failed platinum chemotherapy)	EMA (2023b)
		HER3		
		HER2		
		HER4		
Dacomitinib	TKI	EGFR	EMA-approved—NSCLC (advanced/metastatic, EGFR-activating mutations)	EMA (2021b)
		HER2		
		HER4		
Osimertinib	TKI	EGFR	EMA-approved—NSCLC (advanced/metastatic, EGFR-activating mutations, EGFR exon 19 deletions, EGFR exon 21 (L858R) substitution mutations, and EGFR T790M mutations)	EMA (2023g)
Lapatinib	TKI	EGFR	EMA-approved—breast (HER2+, metastatic, in combination with capecitabine/trastuzumab/aromatase inhibitor)	EMA (2023j)
		HER2		
Neratinib	TKI	EGFR	EMA-approved—breast (HR+, HER2+, and prior trastuzumab therapy)	EMA (2023f)
		HER2		
		HER4		
Tucatinib	TKI	HER2	EMA-approved—breast (HER2+, advanced/metastatic, and >2 prior HER2-targeted therapeutics)	EMA (2023i)
		HER3		
Cetuximab	mAb	EGFR	EMA-approved—colorectal (metastatic, EGFR+, Ras wild-type, single agent/in combination with irinotecan/FOLFOX) and head and neck cancer (squamous, in combination with radiation/platinum chemotherapy)	EMA (2022a)
Panitumumab	mAb	EGFR	EMA-approved—colorectal (metastatic, RAS wild-type, in combination with FOLFOX/FOLFIRI)	EMA (2022b)
Trastuzumab	mAb	HER2	EMA-approved—breast (HER2+) and gastric cancers (HER2+, metastatic, in combination with capecitabine/5-fluorouracil + cisplatin)	EMA (2023c)
Pertuzumab	mAb	HER2	EMA-approved—breast (HER2+, in combination with trastuzumab and chemotherapy)	EMA (2021a)
Ado-trastuzumab emtansine	ADC	HER2	EMA-approved—breast (HER2+, early invasive/advanced/metastatic, prior taxane/HER2-targeted therapeutics)	EMA (2023e)
		Tubulin		
Fam-trastuzumab deruxtecan-Nxki	ADC	HER2	EMA-approved—breast (HER2+, HER2-low, metastatic, prior chemotherapy/HER2-targeted therapeutics), NSCLC (HER2-activating mutation, prior platinum chemotherapy), and gastric cancers (HER2+, advanced, prior trastuzumab therapy)	EMA (2023a)
		Topo-isomerase I		

### 5.1 NOS inhibitors

**FIGURE 4:**
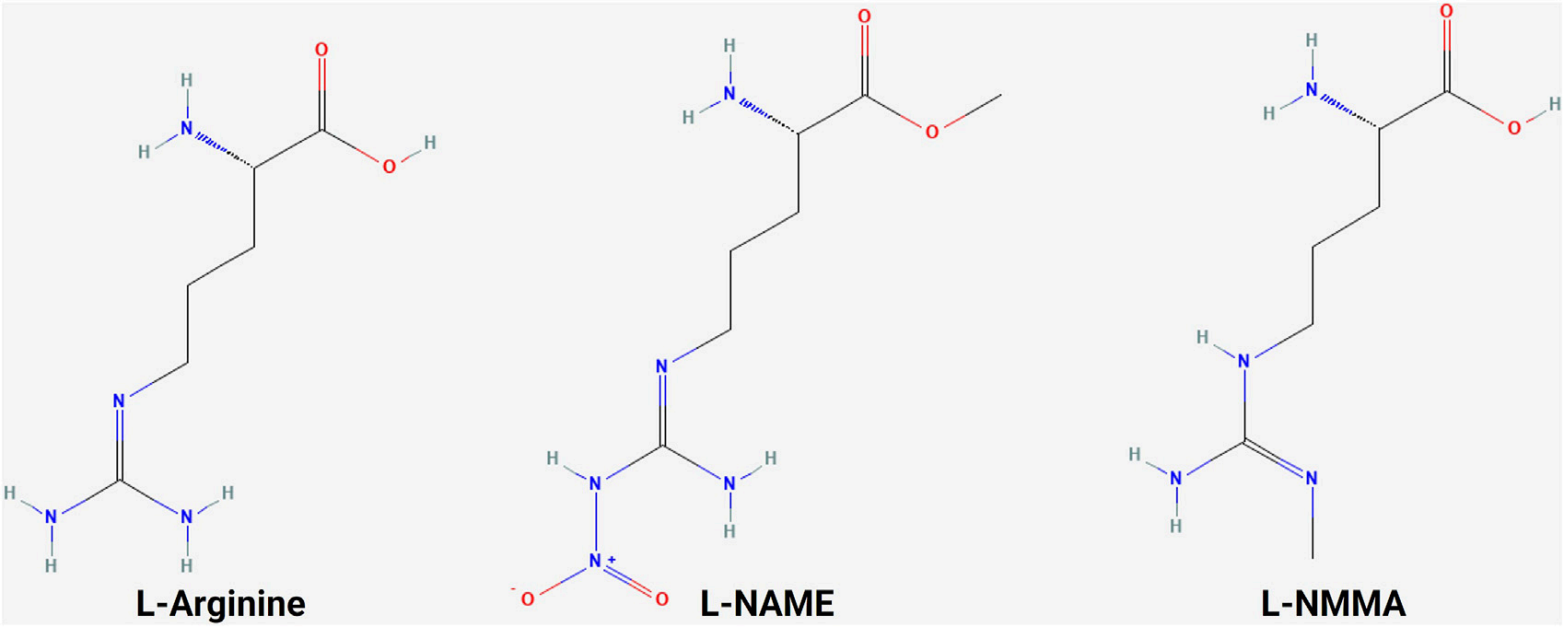
Structure of L-arginine and NOS inhibitors. Chemical structure of L-arginine and NOS inhibitors L-NAME and L-NMMA, showing the structural similarity between the molecules (NCBI, 2024b; NCBI, 2024a; NCBI, 2024c).

A variety of NOS inhibitors, with differing specificities for the three NOS isoforms, are in use by researchers in the NO field. Commonly used NOS inhibitors such as L-NMMA and L-NAME are L-arginine analogs (Figure 4). They bind to NOS’s arginine-binding site, acting as competitive antagonists of the enzyme. These inhibitors have been investigated in clinical trials for various disease states, including cancer (Table 3) (Dao et al., 2021). One such inhibitor, N^G^-monomethyl-L-arginine (L-NMMA), is currently being investigated in the context of triple-negative breast cancer. L-NMMA is a pan-NOS inhibitor that was well-tolerated in an international phase III placebo-controlled trial for cardiogenic shock (TRIUMPH Investigators et al., 2007). L-NMMA, in combination with taxane chemotherapy in locally advanced and metastatic triple-negative breast cancer, was explored in a phase Ib/II clinical trial (Chung et al., 2021). The phase 1 dose-finding wing of the trial recommended a dose of 20 mg/kg for L-NMMA and 100 mg/m^2^ for docetaxel, along with amlodipine and aspirin, to prevent hypertension and thromboembolism, respectively, for the phase II portion. L-NMMA was found to significantly reduce serum nitrates and nitrites, showing successful inhibition of NOS. The overall response was 45.8%, with immune remodeling and a decrease in iNOS expression, following treatment seen in responders. Non-responders had increased levels of circulating fibroblast growth factor (FGF-2), VEGF, IL-8, IL-12p40, IL-1a, and IL-6, which may act as a method of monitoring response. IL-6 can induce STAT3 signaling, a well-known mechanism behind metastasis and proliferation in cancer (Hemmann et al., 1996). Currently, L-NMMA, in combination with nab-paclitaxel and the PI3K inhibitor alpelisib, is being studied in metastatic metaplastic breast cancer in a phase I/II trial (Trial ID: NCT05660083) (The Methodist Hospital Research Institute, 2023). Alternatively, L-NAME, another pan-NOS inhibitor, has been shown to inhibit ERK activation in triple-negative breast cancer *in vitro* (Sciacca et al., 2019). Both STAT3 and ERK signaling are modulated by both NO and HER family signaling, as previously discussed.

**TABLE 3:** NO therapeutics used in clinical trials for cancer.

Drug	Class	Indications	Reference
NO-aspirin (NCX 4016)	NO donor	Phase I—colorectal cancer	Rigas (2009)
L-NMMA	Pan NOS inhibitor	Phase Ib/II—breast cancer (triple negative, advanced/metastatic, in combination with docetaxel, amlodipine, pegfilgrastim, and enteric-coated aspirin)	Chung et al. (2021), Niravath (2023)
RRx-001	Hypoxic NO donor	Phase I—advanced solid tumors	Reid et al. (2015), Reid et al. (2023), Oronsky et al. (2019), Kim et al. (2020), Lee et al. (2021)
	NLRP3 inhibitor	Phase I/II—brain (metastases, in combination with radiation)	
	Nrf2 agonist	Phase II—SCLC (in combination with Etoposide) and colorectal cancer (in combination with irinotecan)	
		Phase III—SCLC (in combination with a platinum doublet)	
Nitroglycerin	NO donor	Phase I—rectal (in combination with 5-fluorouracil and radiation)	Siemens et al. (2009), Dingemans et al. (2015), Illum et al. (2015)
		Phase II—NSCLC (Stage IV, non-squamous, in combination with carboplatin, paclitaxel, and bevacizumab) and prostate cancer (recurrent)	
ASP9853	iNOS inhibitor	Phase I—solid tumors (advanced, in combination with docetaxel)	Luke et al. (2016)

### 5.2 Anti-EGFR therapeutics

The overexpression and mutation of the EGFR have been associated with cancers. EGFR overexpression occurs in gliomas, NSCLCs, and pancreatic adenocarcinomas (Rusch et al., 1997; Wikstrand et al., 1998; Ueda et al., 2004). In gliomas, it is associated with a higher tumor grade and reduced survival (Wikstrand et al., 1998). An EGFR mutation resulting in the loss of its extracellular domain in EGFR type III can cause constitutive activation and has been associated with lung, ovary, and breast cancers (Moscatello et al., 1995). The EGFR has become one of the most popular cancer treatment targets. To date, there are two main drug types for cancer-targeted therapy based on high EGFR expression: tyrosine kinase inhibitors (TKIs) and EGFR monoclonal antibodies. These therapeutic agents have been most successful in the treatment of lung, head and neck, and colorectal cancers.

EGFR TKIs targeting activating EGFR mutations in NSCLC have led to a paradigm shift in the treatment of advanced NSCLC. First- and second-generation TKIs, including gefitinib, erlotinib, afatinib, and dacomitinib, have shown superior overall survival (OS) and progression-free survival (PFS) when compared to platinum-containing chemotherapy (Rosell et al., 2012; Wu et al., 2017; Yoshioka et al., 2019; Sequist et al., 2023). Third-generation osimertinib is an irreversible TKI with significantly prolonged PFS and OS when compared with earlier generations of TKIs (Ramalingam et al., 2020).

EGFR monoclonal antibodies, including cetuximab, panitumumab, nimotuzumab, and necitumumab, exert antitumor activity by competitively binding to different regions of the EGFR extracellular region and inhibiting downstream signaling pathways. In addition, the Fc region of monoclonal antibodies such as cetuximab, panitumumab, and nimotuzumab can bind to the FcR on the surface of different immune cells like natural killer cells, macrophages, and dendritic cells to mediate different innate immune responses (Mazorra et al., 2017). Cetuximab and panitumumab are the most commonly used monoclonal antibodies. When they are combined with chemotherapy, cetuximab and panitumumab improve the response rate and PFS in K-Ras wild-type metastatic colorectal cancer (Cunningham et al., 2004; Karapetis et al., 2008; Douillard et al., 2010). Cetuximab, in combination with radiotherapy, significantly improved overall survival in patients with locoregionally advanced squamous cell carcinoma of the head and neck (Bonner et al., 2010).

Both TKIs and EGFR monoclonal antibodies could interact with the NO pathway, providing potential targets and therapeutic strategies to overcome TKI and monoclonal antibody resistance. Cetuximab, in combination with chemotherapy, has been found to downregulate iNOS and NO levels in colorectal cancer (Benkhelifa et al., 2019). The conjugation of cetuximab with S-nitrosothiol enhances the tumor accumulation of the co-administered antibody (Yoshikawa et al., 2020). Gefitinib is found to act synergistically with NO to induce cell death in metastatic prostate cancer cells (Mimeault et al., 2005). NO-aspirin significantly reduced the number and size of lung tumors *in vivo*, which was linked to reduced levels of EGFR and Akt phosphorylation (Song et al., 2018). A novel hederagenin-NO donor has been found to inhibit proliferation and EGFR kinase activity, even in gefitinib- and osimertinib-resistant NSCLC (Chen et al., 2019).

### 5.3 Anti-HER2 therapeutics

Only HER2 gene amplification with resultant overexpression of the HER2 protein is needed for cellular transformation. HER2 overexpression or amplification leads to ligand-independent dimerization and abnormal downstream signaling. In both mouse fibroblasts and highly transformed tumorigenic cells (Chazin et al., 1992) and in human breast cancer cells, HER2 overexpression results in increased tumorigenicity (Benz et al., 1992). It is found in approximately 25% of breast cancers and is historically associated with aggressive disease and a poor prognosis (Slamon et al., 1989). The discovery that HER2 overexpression was associated with an extremely poor outcome in breast cancer led to the development of the monoclonal antibody trastuzumab and many other agents later, which revolutionized the outcome of patients with HER2-positive breast cancer.

Similar to EGFR-targeted therapies, anti-HER2 therapeutics also include TKIs, such as lapatinib, neratinib, pyrotinib, and tucatinib, and monoclonal antibodies, like trastuzumab and pertuzumab (Cho et al., 2003; Franklin et al., 2004; Arcila et al., 2012). Trastuzumab was the first humanized monoclonal antibody developed that achieved remarkable success. Another successfully developed monoclonal antibody is pertuzumab. Although trastuzumab binds to the extracellular domain IV of HER2, pertuzumab binds to the extracellular domain II, which prevents HER2 heterodimerization with EGFR, HER3, and HER4. Combinations of trastuzumab and pertuzumab provide complementary mechanisms of action and were proven superior to single-agent trastuzumab in neoadjuvant, adjuvant, and metastatic settings in breast cancer (Nahta et al., 2004; Gianni et al., 2012; Swain et al., 2020). The success of targeting HER2 as a therapeutic strategy was seen in other malignancies that overexpress HER2. Trastuzumab, in combination with chemotherapy, improved OS in patients with HER2-positive gastric or gastro-esophageal junction cancer and endometrial cancer (Bang et al., 2010; Fader et al., 2020).

TKIs are small molecules that target the intracellular catalytic kinase domain of HER2, competing with ATP, blocking phosphorylation and the activation of downstream signaling cascades. Because of their small molecular size, some TKIs have shown the ability of penetrating the blood–brain barrier and anti-tumor efficacy in the CNS. Lapatinib monotherapy and combination therapy demonstrated some efficacy in patients with HER2-positive breast cancer and CNS diseases (Geyer et al., 2006; Lin et al., 2009). Most recently, a newer-generation TKI tucatinib, in combination with capecitabine and trastuzumab, for the first time, demonstrated clinically meaningful benefits in patients with HER2-positive active brain metastases (Murthy et al., 2020).

Recently, the anti-HER2 therapeutics have been expanded to include antibody–drug conjugates (ADCs) such as ado-trastuzumab emtansine (T-DM1) and fam-trastuzumab deruxtecan-nxki (T-DXd). ADCs contain a tumor-targeting antibody covalently bound to a cytotoxic drug (payload) via a synthetic linker. The ADC is directed to cancer cells expressing the target on the cell surface, followed by the internalization of the ADC and release of the cytotoxic payload, resulting in tumor cell death. T-DM1 was the first anti-HER2 ADC developed that contains DM1, a maytansine derivative, as a payload with a drug-to-antibody ratio of 3.5. T-DM1 prolonged PFS and OS in patients with HER2-positive breast cancer in the metastatic setting (Verma et al., 2012) and in the adjuvant setting in patients with residual disease after neoadjuvant treatment (von Minckwitz et al., 2019). T-DXd is a newer ADC that has deruxtecan as a payload and a drug-to-payload ratio of 8. T-DXd demonstrated unprecedented improvement in PFS when compared head-to-head with T-DM1 in patients with metastatic HER2-positive breast cancer, leading to its FDA approval (Cortés et al., 2022).

In addition to HER2 gene amplification, HER2 mutation can also activate the downstream signaling pathway and drive tumorigenesis. In contrast to HER2 overexpression, HER2 mutations are identified in a wider variety of solid organ malignancies. HER2 mutations can be detected in up to 15%–19% of prostate neuroendocrine tumor and bladder cancer; 3%–6% of colorectal, gastric, and esophageal cancers; and less than 3% in breast and lung cancers (Connell and Doherty, 2017). Despite the success of monoclonal antibodies and TKIs in HER2 overexpressed cancer, they have only shown minor benefits in HER2-mutated malignancies. In contrast, ADCs, especially T-DXd, have shown encouraging results in HER2-mutated advanced lung cancer and gastroesophageal cancer that led to FDA approvals (Shitara et al., 2020; Li et al., 2022).

Despite the success in the development of anti-HER2 therapeutics, resistance inevitably happens in many patients. In addition to mutations like ΔHER2 (Siegel et al., 1999), which results in a higher level of homodimer formation and phosphorylation, altered s-nitrosation in HER2+ breast cancer through GSNOR inhibition has also been identified to induce trastuzumab resistance (Cañas et al., 2016). The interactions between gefitinib and NO discussed above are also relevant in the context of HER2 as the response to gefitinib in breast cancer was found to be independent of EGFR expression but influenced by HER2 overexpression (Campiglio et al., 2004).

### 5.4 Anti-HER3 therapeutics

The presence of HER3 has been documented in multiple cancers (Ciardiello et al., 1991; Rajkumar et al., 1993; 1996; Friess et al., 1995; Simpson et al., 1995; Bobrow et al., 1997; Leung et al., 1997; Yi et al., 1997; Slesak et al., 1998; Reschke et al., 2008; Ocana et al., 2013; Zhang et al., 2015) and linked to both treatment failure and drug resistance in breast, prostate, ovarian, and NSCLC (Holbro et al., 2003; Engelman et al., 2007; Mills and Yarden, 2010; Jathal et al., 2011). The upregulation of HER3 expression or signaling is associated with resistance to HER2 inhibitors in HER2-overexpressed breast cancer and to EGFR inhibitors in lung cancer; HER3 mutations have been reported as an oncogenic driver in colon and gastric cancers. These findings suggest that HER3 plays a pivotal role in the upregulation of tumor growth and drug resistance (Jacob et al., 2018). Despite the role of HER3 in mediating resistance, the inhibition of HER3 with either anti-HER3 monoclonal antibodies or in combination with anti-EGFR, anti-HER2, or chemotherapy only provided marginal clinical benefit. An increased incidence of diarrhea was also observed when anti-HER3 therapies were combined with anti-HER2 therapies (Aurisicchio et al., 2012; McDonagh et al., 2012; Huang et al., 2013).

The interactions between gefitinib and NO discussed above are also relevant in the context of HER3 as gefitinib induces the formation of inactive EGFR/HER2 and EGFR/HER3 dimers, along with inhibiting the formation of active HER2/HER3 dimers in HER2-amplified breast cancer (Anido et al., 2003).

### 5.5 Anti-HER4 therapeutics

HER4 has been linked to various cancers, such as breast, colorectal, lung, hepatocellular, prostate, bladder, ovarian, endometrial, and glioblastoma (Edwards et al., 2006; Memon et al., 2006; Ejskjaer et al., 2007; Sundvall et al., 2008; de Wit et al., 2013; Liu et al., 2017; Saglam et al., 2017; Zhang et al., 2017; Donoghue et al., 2018). However, the role of HER4 is less straightforward.

HER4 expression in breast cancer has been linked to improved outcomes in ER + disease due to its anti-proliferative activity (Tovey et al., 2004). HER4 expression has also been linked to improved sensitivity to trastuzumab (Portier et al., 2013). In bladder and hepatocellular carcinoma, decreased HER4 expression is linked to a poor prognosis (Memon et al., 2006; Liu et al., 2017). On the other hand, in lung cancer, specific HER4 polymorphisms are linked to a higher risk of developing the disease (Zhang et al., 2017). In glioblastoma and endometrial cancer, HER4 expression is not correlated with survival (Ejskjaer et al., 2007; Donoghue et al., 2018). Afatinib and allitinib are kinase inhibitors that act on EGFR, HER2, and HER4 (Solca et al., 2012; Zhang et al., 2014). Recently, novel imidazothiazole derivatives were found to act as specific HER4 kinase inhibitors (Zaraei et al., 2021), which might help delineate the role of HER4 in various solid tumor malignancies.

## 6 Conclusion

Since its discovery in the 1960s, the role of the EGFR and its sister receptors in cancer has become increasingly apparent. Aberrant EGFR and HER2 signaling is widely accepted to drive disease progression and result in poorer patient outcomes. Nitric oxide has been proposed as an alternative activator of the HER family and may play a role in this aberrant activation as high iNOS expression has been associated with outcomes in the ER-negative and triple-negative breast cancer setting. NO is a promising druggable target with widespread involvement in oncogenic signaling through the induction of EGFR phosphorylation and s-nitrosation. Due to the structural similarity between the EGFR and the rest of the receptor family, NO is also likely to induce their phosphorylation and s-nitrosation. Signaling molecules downstream of the HER family, Ras and PTEN, also undergo s-nitrosation, resulting in increased Ras and PI3K signal transduction, further demonstrating the role of NO in signaling associated with these receptors. Additionally, NO has been shown to reduce EGFR signaling and act synergistically with gefitinib, an EGFR tyrosine kinase inhibitor, in prostate and lung cancers, while altered s-nitrosation in HER2+ breast cancer is linked to trastuzumab resistance. This demonstrates a role for NO in the treatment of patients with tumors driven by HER family signaling. However, further research is needed to fully unravel the role of NO in the activation of HER family signaling and its impact on treatment outcomes as research to date has predominantly focused on the EGFR.
